# *Lactococcus lactis* strain Plasma activates plasmacytoid dendritic cells and mitigates common cold-like symptoms in healthy adults: a meta-analysis of individual participant data

**DOI:** 10.3389/fimmu.2025.1696989

**Published:** 2025-11-06

**Authors:** Yukiko Kato, Keiko Kobayashi, Yui Kuramochi, Toshio Miyata, Yusuke Ushida, Kohsuke Hayamizu

**Affiliations:** 1 Institute of Health Sciences, Kirin Holdings Company, Limited, Fujisawa, Japan; 2 Laboratory of Food Chemistry, Yokohama University of Pharmacy, Yokohama, Japan; 3 Center for Advanced Biomedical Sciences, Waseda University, Tokyo, Japan; 4 Mih Clinic, Tokyo, Japan

**Keywords:** *Lactococcus lactis* strain Plasma, lactic acid bacteria, probiotics, plasmacytoid dendritic cell, common cold, meta-analysis

## Abstract

**Background:**

Plasmacytoid dendritic cells (pDCs) are a type of immune cell that play a crucial role in the defense against viral infection. Multiple randomized controlled trials (RCTs) have reported that oral intake of *Lactococcus lactis* strain Plasma (LC-Plasma) activates pDCs and reduces cold-like symptoms. This study conducted a meta-analysis to comprehensively evaluate the effects of oral LC-Plasma intake on pDC activation and cold-like symptoms by comparing healthy adults.

**Method:**

This study targeted RCTs that examined the effects of oral intake of LC-Plasma or placebo food products on pDC activation or common cold-like symptoms in healthy adult males and females. Data sources included PubMed, Cochrane Library, J-Dream III, UMIN-CTR, and the International Clinical Trials Registry Platform, with searches conducted up to June 21^st^, 2024. The primary outcome evaluated pDC activation, and the secondary outcome evaluated the subjective evaluation of common cold-like symptoms. A quantitative synthesis was performed by meta-analysis using random-effects models. Two authors independently assessed risk of bias using the revised Cochrane risk of bias tool (RoB2).

**Results:**

Among the eight RCTs that met the eligibility criteria for this study, individual participant data (IPD) were obtained from seven. Since we were unable to obtain IPD from the remaining study, we integrated data from this study based on its final report. The meta-analysis in this study, conducted using data from 619 participants, revealed that the expression levels of pDC activation markers, CD86 and HLA-DR, were significantly maintained during LC-Plasma intake when compared to the placebo control group (CD86: SMD = 0.37, 95% CI (confidence interval): 0.17 to 0.57; HLA-DR: SMD = 0.47, 95% CI: 0.21 to 0.73). In addition, LC-Plasma intake significantly reduced the cumulative number of days per 28 days of cough and feverishness compared to the control group, according to the meta-analysis using data from 555 participants (Cough: MD=-0.69, 95%CI: -1.24 to -0.14; Feverishness: MD=-0.26, 95%CI: -0.52 to -0.0038).

**Conclusion:**

This study is the first to present, through integrated analyses using identical analytical conditions, that daily intake of LC-Plasma may help sustain pDC activation and could be useful in reducing cough and feverishness, typical cold-like symptoms.

**Systematic Review Registration:**

https://www.umin.ac.jp/, identifier UMIN000054706.

## 1 Introduction

The common cold is one of the most common contagious diseases in humans and is caused primarily by viruses. It is associated with the expression of local symptoms such as cough, runny nose, and sore throat and may also involve systemic symptoms such as fever (1–3). Usually, common cold symptoms are mild, and the patient recovers naturally; thus, no effective vaccine or therapeutic drugs have been developed. However, the common cold remains a social problem due to its high prevalence. It often lowers quality of life (QOL) for patients and causes considerable economic losses due to both a reduced workforce resulting from absenteeism or decreased on-the-job productivity. A study in the United States reported that contagious respiratory infections caused by viruses, not involving the influenza virus, resulted in approximately 22.5 billion dollars in productivity loss annually (4). For these reasons, it is important to maintain daily routines that help prevent contracting the common cold (5, 6).

Plasmacytoid dendritic cells (pDCs) are a minor subset, accounting for approximately 0.1% to 0.5%, of human peripheral blood mononuclear cells (PBMCs) (7). Despite that, they are immune cells that play key roles in the defense against viral infections. When pDCs detect viruses or foreign objects through toll-like receptors, TLR7 and TLR9, they produce large amounts of type I interferon (IFN) (7–12). Type I IFN directly prevents viral infection by inducing the expression of enzymes that inhibit viral replication, as well as RNA-degrading enzymes that break down viruses (13–15). Furthermore, they enhance both innate immunity and acquired immunity by activating the antiviral functions of natural killer cells, myeloid DCs, B cells, and T cells (13–15). In addition, activation of pDCs via toll-like receptors induces the upregulation of major histocompatibility complex class II molecules (such as HLA-DR; Human Leukocyte Antigen - DR isotype) and costimulatory molecules (such as Cluster of Differentiation (CD)86 and CD80), enabling them to present antigens to CD4^+^ T cells (7, 11, 16, 17). With these functions, pDCs provide defense against infections caused by viruses that lead to the common cold (such as rhinovirus and coronavirus) (5, 8).

*Lactococcus lactis* strain Plasma (LC-Plasma) was discovered through *in vitro* studies using dendritic cells derived from mouse bone marrow cells and was identified as a unique lactic acid bacterium that directly activates pDCs. Both live and heat-killed LC-Plasma have been reported to induce IFN-α production (18). In 2013, long-term consumption of yogurt containing LC-Plasma was reported to increase pDC activity in peripheral blood compared to a control group. In addition, during the period of yogurt consumption, the LC-Plasma group reported fewer days of common cold-like symptoms compared to the control group during the period of yogurt consumption compared to the control group (19). Furthermore, a clinical study reported in 2015 demonstrated that, compared to a control group, PBMCs obtained from participants who consumed yogurt containing LC-Plasma exhibited a significantly enhanced antiviral response when stimulated with inactivated influenza virus (20). In addition, multiple randomized controlled tests (RCTs) confirmed that consuming LC−Plasma significantly enhanced pDC activity and alleviated cold-like symptoms (21, 22). Meanwhile, no integrated efficacy study, including a quantitative assessment of the effects of LC−Plasma on activating pDCs and cold-like symptoms, has been conducted. In addition, individual RCTs used different methods to assess daily physical conditions (common cold-like symptoms), and the cold-like symptoms on which LC-Plasma showed efficacy varied among studies.

Our study aimed to comprehensively verify the effects of oral intake of LC-Plasma on pDC activity and prevention of the common cold, including quantitative evaluation, by conducting a meta-analysis using Individual Participant Data (IPD).

## 2 Methods

### 2.1 Study procedures

The study protocol and documents of this study, including the informed consent form, were approved by the Human Research Ethics Committee of Kirin Holdings Company, Limited (Tokyo, Japan) in accordance with the ethical guidelines outlined in the Declaration of Helsinki and the Ethical Guidelines for Life Science and Medical Research Involving Human Subjects (approval number: 2024-002). This study was prospectively registered with the UMIN Clinical Trials Registry (UMIN-CTR) (https://www.umin.ac.jp/, UMIN000054706) and was conducted in accordance with the Preferred Reporting Items for Systematic Review and Meta-Analysis (PRISMA) guidelines (23, 24).

### 2.2 Search strategy and eligibility criteria

Eligible studies were identified based on the PICOS (Population, Intervention, Comparator, Outcomes, Study design) criteria, defined as follows: P) healthy adult men and women (individuals without any diseases) (minors under 18 years of age, pregnant or lactating women, including those planning pregnancy, were excluded), I) oral intake of foods or beverages containing LC-Plasma or lactic acid bacteria of the same strain as LC-Plasma, C) oral intake of foods or beverages not containing LC-Plasma or lactic acid bacteria of the same strain as LC-Plasma (placebo), O) pDC activity and daily physical condition (subjective evaluation of cold-like symptoms), S) Randomized controlled trials (RCTs). Exclusion criteria encompassed: (1) Studies including participants with existing diseases; (2) Studies with substantial deviations from the defined PICOS criteria; (3) Reviews, books, and other non-primary literature; (4) Studies published in languages other than English or Japanese; (5) Non-peer-reviewed publications; (6) Studies conducted without appropriate ethical approval.

A comprehensive search was conducted across three literature databases, PubMed, Cochrane Library and J-Dream III (Japanese database), as well as two clinical trial registries, the UMIN-CTR and the Clinical Trials Registry Platform (ICTRP), to obtain an initial list of pertinent studies. All relevant studies published up to June 21^st^, 2024 were included without any publication date restrictions. No hand researching was performed. To ensure comprehensiveness, the literature search excluded terms related to study design or outcomes for narrowing results. Instead, the search strategy employed terms related to LC-Plasma and strains corresponding to LC-Plasma. The detailed search strategy is provided in **Supplementary Table 1**. The literature search was conducted by a single reviewer.

During the primary screening, studies were selected based on titles and abstracts, followed by a secondary screening involving full-text review. Two reviewers independently conducted the screening process and documented reasons for excluding ineligible studies. Any disagreements between reviewers were resolved through discussion with a third reviewer.

### 2.3 Data collection processes

IPD were requested to the authors of each eligible study. The data were requested to be provided in a format convenient for the authors, with personally identifiable information such as names removed and replaced by unique identifiers. The received data were converted into formats suitable for analysis using statistical software EZR (Easy R). In the case that IPD were unavailable, relevant information was extracted from the published articles or final study reports. The results of the included studies were grouped according to outcomes. The integrity of the IPD was confirmed by reproducing the analytical methods reported in the publications.

### 2.4 Risk of bias assessment

Two reviewers independently assessed the risk of bias for each outcome of the selected studies using the risk of bias assessment tool (RoB2) (25). Any discrepancies in the assessments were resolved through discussion with a third reviewer. Exclusion based on risk of bias results was not planned.

### 2.5 Definition of outcomes

The primary endpoint of this study was the change in expression levels of CD86 or HLA-DR, which are pDC activation markers, measured before and after the intervention. The observation data from individual participants were correlated before and after the intervention; therefore, the amount of change measured between the pre- and post-intervention values was designated as the variable for the analysis. When multiple analysis points were set, the point at the end of the intervention period was adopted as the sole variable for analysis. Among the study participants, those who were analyzed for pDC activity in their respective studies were included as subjects for analysis in this study.

This study adopted objective evaluations of daily physical conditions (cold-like symptoms) as the secondary endpoint, and the cumulative number of days with cold-like symptoms and their severities were used as analytical indices. This study adopted symptoms that narrowly define the common cold (i.e., runny nose, sore throat, and cough), along with “feverishness” which is an indicator of systemic symptoms, as variables for evaluation (1, 2, 26–29). For feverishness, this study used items categorized as “fever” in the 2021 study by Khor and those categorized as “feverishness” in the remaining three studies (19, 20, 30). Since the frequency of common cold-like symptoms depends on the length of the observation period, this study included only those studies with an intervention period of four weeks or longer as subjects for analysis. Study participants who were evaluated for their common cold-like symptoms in their respective studies were included as subjects for analysis in this study. In all studies, participants self-reported individual symptoms daily using a five-level scoring system (Normal = 1, Slight = 2, Mild = 3, Moderate = 4, and Severe = 5). If daily logs had missing measurement values, the score recorded on the previous day was used as a substitute. Since cold symptoms usually peak on the third day, the cumulative number of days during the observation period on which symptoms (either Slight, Mild, Moderate, or Severe) appeared for three or more consecutive days was adopted as the “cumulative number of days of cold-like symptom” for each symptom (26, 31). Since the common cold is narrowly defined as the simultaneous occurrence of three symptoms: runny nose, sore throat, and cough, days with at least two of these symptoms for three or more consecutive days were defined as “common cold onset” days (26–29). The cumulative number of common cold days was converted into the number of days per 28 days and used for integration. The severity was calculated based on the average score over the observation period. This study aimed to examine the effect of the test food product in preventing the common cold. Thus, individuals who expressed cold-like symptoms on the first day of intervention (study subjects who showed symptoms for three consecutive days starting from the first day of intervention) were excluded from the analysis of the cumulative number of days of each symptom and their severity (defined as “per-protocol of MA”).

### 2.6 Statistical analysis

Statistical analyses were executed using EZR Version 1.52 (R Version 4.0.2) (32). The mean and standard deviation (SD) of change of CD86 and HLA-DR expression before and after intervention were calculated for each study using IPD. For the study in which IPD could not be obtained, changes in the mean and SD were calculated from the difference between the values observed before and after intervention, as reported in the final report. Changes in SD were calculated using the formula: SD*_change_* =√(SD^2^*_before_*+SD^2^*_after_*−2×Corr×SD*_before_*×SD*_after_*), assuming a correlation coefficient R = 0.5 (33). SD*_before_* and SD*_after_* were reported in the final report. The SD of the observed values before and after the intervention were as reported in the final report. The amount of change measured between the pre- and post-intervention values of pDC activity were expressed using standardized mean difference (SMD) because pDC activities were measured using different flow cytometer devices and reagents (antibodies) across the included studies. An SMD greater than 0 indicated the beneficial effects of the LC-Plasma intervention. For secondary outcomes, the cumulative days of occurrence and mean scores of each common cold-like symptom were analyzed using mean difference (MD). An MD less than 0 indicated the beneficial effects of the LC-Plasma intervention. Quantitative synthesis was performed by two step IPD meta-analysis for each outcome using random-effects models, and forest plots were generated. Study heterogeneity was assessed using the I² statistics, and the factors modifying the effects of LC-Plasma intervention were investigated when heterogeneity was high. Statistical analyses were performed based on the pooled effect sizes comparing the intervention and control groups, accompanied by their respective 95% confidence intervals. As a subgroup analysis, the present study examined differences in the effects of the intervention depending on the presence of pollen allergy symptoms, which present similarly to common cold symptoms. The analysis also identified whether a study conducted the intervention during the pollen dispersal season without excluding individuals with pollen allergy based on exclusion criteria.

### 2.7 Assessment of the credibility of evidence

The quality of evidence was assessed using the five GRADE framework (risk of bias, consistency of effect, imprecision, indirectness, and others including publication bias) and categorized as “high,” “moderate,” “low,” or “very low” quality by two investigators, collaboratively (34).

## 3 Results

### 3.1 Study selection and IPD obtained

**Figure 1:**
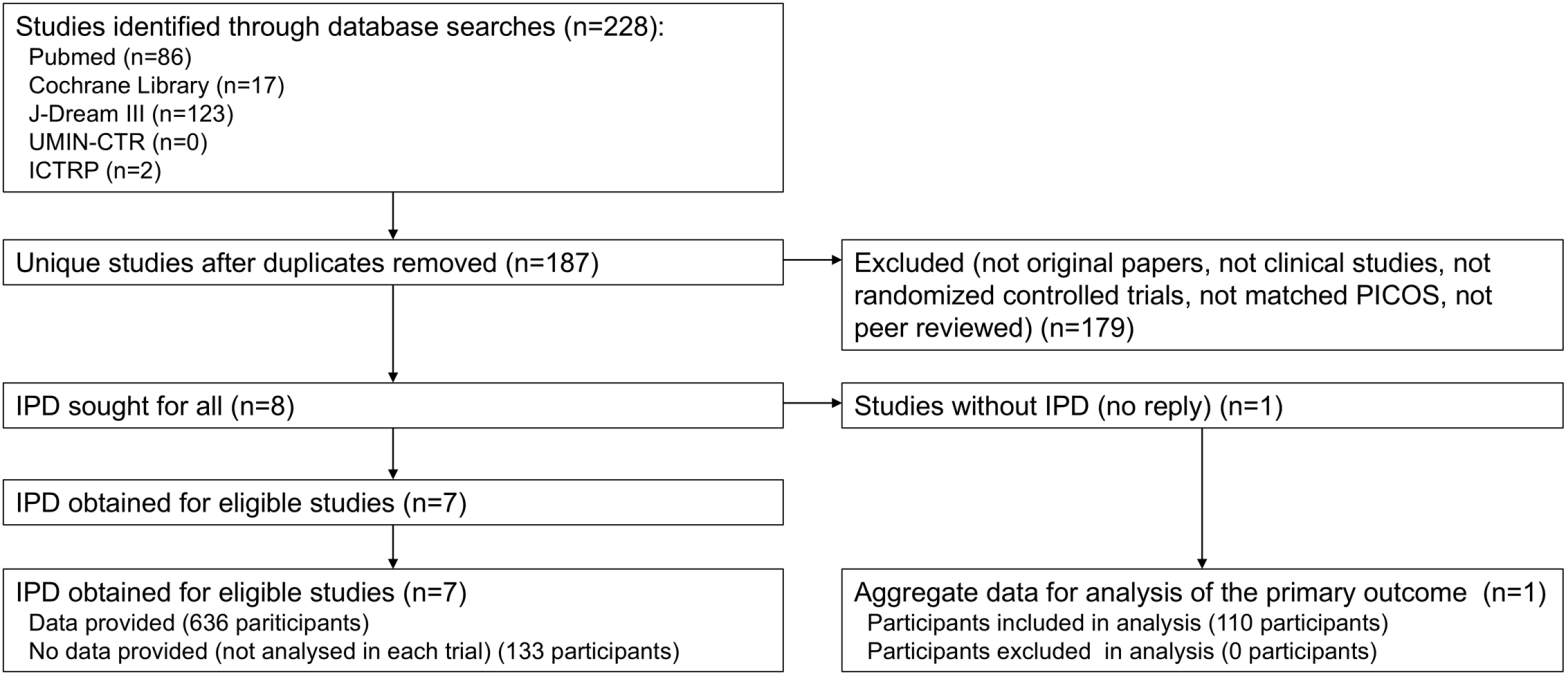
Flow of the process of study selection.

The selection process is presented in a PRISMA flow diagram (**Figure 1**). Our initial search across all databases identified 228 potentially relevant records. After removal of duplicates, 14 studies remained for title and abstract screening. Following full-text review, eight studies were incorporated into this meta-analysis (19–22, 30, 35–37). IPD were sought and obtained from seven studies. For one study (22), IPD were not obtained due to non-response from the author holding the data; however, summary statistics for the primary outcome were obtained from another author. Ultimately, IPD from 636 participants across seven studies were obtained, while IPD for 131 participants excluded from analysis in each study were unavailable. Additionally, summary statistics for 110 participants (primary outcome only) were obtained from one study. For the seven studies with IPD, analyses identical to those reported in the publications were conducted using the IPD, confirming consistency with the published results.

### 3.2 Study and participant characteristics

**Table 1** presents the characteristics of eligible studies and their participants. This meta-analysis included eight RCTs. Studies were conducted in Japan or Malaysia and enrolled participants of both sexes from 18 to 64 years of age. Two studies were conducted using test foods containing live LC-Plasma, while six studies employed test foods containing heat-killed LC-Plasma for the intervention. In all studies, the daily intake was set at 1×10^11^ cells of LC-Plasma. The intervention periods ranged from 13 to 84 days. Among the studies with an intervention period of four weeks or longer, six studies assessed common cold-like symptoms, and among these, IPD were obtained from five studies. pDC activity was designated as the primary outcome in three studies, and as a secondary outcome in another three studies, with one study not reporting this information. Six studies identified common cold-like symptoms as primary outcomes, while two studies did not specify. All studies were funded by Kirin Company, Limited (now Kirin Holdings Company, Limited) or Kirin Holdings Company, Limited.

**Table 1:** Characteristics of the eight eligible studies and their participants.

Study	Setting	Participants (male: female)	Age (yr), mean(SD)		Intake duration [days]	Active food	Study design	Outcome of pDC activity (outcome type)	Outcome of common cold (outcome type)	Sponsor	Data
			LC-Plasma	Control							
2013_Sugimura (19)	Japan	Healthy adults (31:7)	39.1 (8.2)	39.6 (8.2)	28 (August)	Live LC-Plasma in yogurt (1×10^11^ cfu/day)	RCT	CD86, HLA-DR (not referred)	Cumulative incidence days by self-report (not referred)	Kirin Co., Ltd.**	IPD
2015_Sugimura (20)	Japan	Healthy adults (92:121)	45.5 (7.6)	45.1 (7.6)	70 (January to March)	Live LC-Plasma in yogurt (1×10^11^ cfu/day)	RCT	CD86 (Secondary)	Cumulative incidence days by self-report (Primary)	Kirin Co., Ltd.**	IPD
2015_Suzuki (21)	Japan	Healthy adults (50:50)	43 (10.5)	44 (9.6)	56 (February to March)	Heat-killed LC-Plasma in beverage (1×10^11^ cells/day)	RCT	CD86, HLA-DR (Primary)	Cumulative incidence days by self-report (Primary)	Kirin Co., Ltd.**	IPD
2016_Shibata (22)	Japan	Healthy adults (122:274)	21.6 (0.3)	21.3 (0.3)	84 (December to March)	Heat-killed LC-Plasma in capsules (1×10^11^ cells/day)	RCT	HLA-DR (Secondary)	Incidence rates and severity by self-report (Primary)	Kirin Co., Ltd.**	Summary statistics
2017_Fujii (30)	Japan	Healthy adults (61:46)	38.9 (9.6)	38.4 (9.2)	28 (April to May)	Heat-killed LC-Plasma in capsules (1×10^11^ cells/day)	RCT	CD86 (Secondary)	Severity by self-report (Primary)	Kirin Co., Ltd.**	IPD
2018_Komano (35)	Japan	Healthy university students (50:0)	20.8 (0.8)	20.5 (0.8)	13 (January to February)	Heat-killed LC-Plasma in capsules (1×10^11^ cells/day)	RCT	CD86, HLA-DR (Primary)	Cumulative incidence days after high intensity exercise (Primary)	Kirin Co., Ltd.**	IPD
2021_Khor (36)	Malaysia	Healthy adults (27:76)	36.1 (13.1)	37.7 (14.3)	56* (February to April)	Heat-killed LC-Plasma in tablets (1×10^11^ cells/day)	RCT	–	Presentation and severity of Dengue fever like symptoms (not referred)	Kirin Holdings Co., Ltd.	IPD
2023_Komano (37)	Japan	Healthy university students (37:0)	19.9 (1.2)	19.8 (1.3)	14 (August to September)	Heat-killed LC-Plasma in capsules (1×10^11^ cells/day)	RCT	CD86, HLA-DR (Primary)	Cumulative incidence days after high intensity exercise (Primary)	Kirin Holdings Co., Ltd.	IPD

*Due to the impact of the COVID-19 pandemic, only the 8-week intervention period was included in the analysis, though a 16-week intervention had been planned.

**Kirin Co., Ltd. is Current Kirin Holdings Co., Ltd. LC-Plasma, *Lactococcus lactis* strain Plasma; pDC, plasmacytoid dendritic cells; HLA-DR, Human Leukocyte Antigen - DR isotype; CD86, costimulatory molecule, Cluster of Differentiation 86

### 3.3 Risk of bias within eligible studies

**Supplementary Figure 1** provides results of the risk of bias assessment. All eight studies were assessed as having some concerns for pDC activity markers CD86 and HLA-DR (**Supplementary Figures 1A, B**). For cumulative number of days with sore throat, runny nose, and cough, all five studies consistently indicated a high risk of bias (**Supplementary Figures 1C–E**). For the cumulative number of days with feverishness, three out of four studies were assessed as having some concerns, while one study was assessed as being at high risk (**Supplementary Figure 1F**). One out of four studies demonstrated some concerns for the cumulative number of days with common cold, and the other three were assessed as being high risk (**Supplementary Figure 1G**). The main reason for the high risk of bias between studies in the cumulative days of sore throat, runny nose, and cough was that participants with each symptom that persisted for more than three days from the start of intervention were excluded from this meta-analysis, so not all assigned participants were included in the analysis. This resulted in a high risk assessment in domain 2. Consistent with the protocol, no studies were excluded for the analyses based on the risk of bias assessment results.

### 3.4 Results of syntheses

#### 3.4.1 Primary outcome: pDC activity

**Figure 2:**
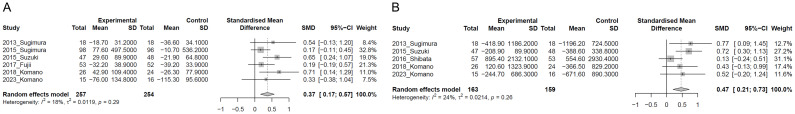
Forest plots of plasmacytoid dendritic cell (pDC) activity. **(A)** CD86 expression on pDC; **(B)** HLA-DR expression on pDC. “Experimental” in the figure indicates the results of LC-Plasma intervention.

**Figure 2** presents the results of the two-step IPD meta-analysis of primary outcome. CD86 expression levels on pDCs were evaluated in six studies. The mean and SD of change of CD86 expression before and after intervention were calculated for each study using IPD. LC-Plasma intake resulted in a statistically significant maintained higher CD86 expression compared to the control group (SMD = 0.37, 95%CI: 0.17 to 0.57, p<0.001, I^2^ = 18%; 511 participants in six studies) (**Figure 2A**). HLA-DR expression levels on pDCs were evaluated in five studies. The mean and SD of change of CD86 expression before and after intervention were calculated for each study using IPD from four studies. For one study (2016_Shibata), from which IPD could not be obtained, the mean change was calculated from the difference in mean observed values before and after the intervention and the SD of the change was estimated using the SD of the observed values before and after the intervention as reported in the final report. LC-Plasma intake resulted in a statistically significant maintained higher HLA-DR expression compared to the control group (SMD = 0.47, 95%CI: 0.21 to 0.73, p<0.001, I^2^ = 24%; 322 participants in five studies) (**Figure 2B**).

To explore reasons for heterogeneity, we conducted subgroup analysis without the study from which IPD could not be obtained (2016_Shibata). As a result, LC-Plasma intake resulted in a statistically significant maintained higher HLA-DR expression compared to the control group (SMD = 0.63, 95%CI: 0.35 to 0.90, p<0.001, I^2^ = 0%; 212 participants in four studies) (**Supplementary Figure 2**). In the meta-analysis including the 2016_Shibata study, the heterogeneity was I² = 24%, whereas in the meta-analysis excluding the 2016_Shibata study, the heterogeneity was I² = 0%, suggesting that the 2016_Shibata study was the source of heterogeneity. The inability to obtain IPD from this study may have contributed to the observed heterogeneity.

#### 3.4.2 Secondary outcomes

##### 3.4.2.1 Cumulative number of days of common cold-like symptoms

**Figure 3:**
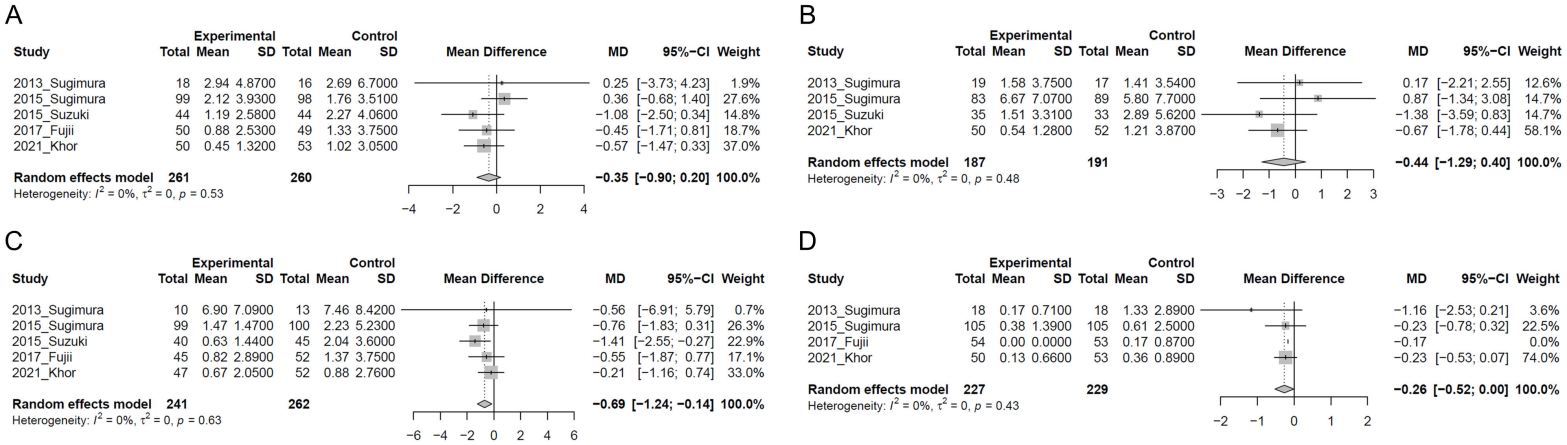
Forest plots of cumulative number of common cold-like symptoms. **(A)** Sore throat; **(B)** Runny nose; **(C)** Cough; **(D)** Feverishness.

**Figure 3** presents the results of the two-step IPD meta-analysis of cumulative number of days of common cold-like symptoms. In the LC-Plasma group, no significant change was observed in the cumulative number of days of sore throat compared to the control group (MD=-0.35, 95%CI: -0.90 to 0.20, p=0.209, I^2^ = 0%; 521 participants in five studies) (**Figure 3A**). Similarly, in the LC-Plasma group, no significant change was observed in the cumulative number of days of runny nose compared to the control group (MD=-0.44, 95%CI: -1.29 to 0.40, p=0.306, I^2^ = 0%; 378 participants in four studies) (**Figure 3B**). LC-Plasma intake significantly reduced the cumulative number of days per 28 days with cough compared to the control group (MD=-0.69, 95%CI: -1.24 to -0.14, p=0.013, I^2^ = 0%; 503 participants in five studies) (**Figure 3C**). Additionally, LC-Plasma intake also significantly decreased the cumulative number of days per 28 days with feverishness compared to the control group (MD=-0.26, 95%CI: -0.52 to -0.0038, p=0.047, I^2^ = 0%; 456 participants in four studies) (**Figure 3D**).

##### 3.4.2.2 Severity of common cold-like symptoms

We evaluated the effect of LC-Plasma intake in severity of common cold-like symptoms in a two-step IPD meta-analysis (**Supplementary Figure 3**). In the LC-Plasma group, no significant change was observed in the severity of sore throat compared to the control group (MD=-0.001, 95%CI: -0.041 to 0.022, p=0.548, I^2^ = 0%; 521 participants in five studies) (**Supplementary Figure 3A**). Similarly, in the LC-Plasma group, no significant change was observed in the severity of runny nose compared to the control group (MD=-0.018, 95%CI: -0.065 to 0.029, p = 0.447, I^2^ = 0%; 378 participants in four studies) (**Supplementary Figure 3B**). In the severity of feverishness, no significant change was observed in the LC-Plasma group compared to the control group (MD=-0.013, 95%CI: -0.031 to 0.006, p= 0.177, I^2^ = 0%; 456 participants in four studies (**Supplementary Figure 3D**). LC-Plasma intake also significantly mitigated the severity of cough score during the intervention compared to the control group (MD=-0.049, 95%CI: -0.083 to -0.014, p=0.006, I^2^ = 0%; 503 participants in five studies) (**Supplementary Figure 3C**).

#### 3.4.3 Subgroup analyses

No heterogeneity in the results of this meta-analysis was observed for the cumulative duration of each symptom. However, the 2015_Sugimura study showed a higher number of days with runny nose compared to other studies, prompting a review of its study conditions. It was found that the intervention in the 2015_Sugimura study was conducted during the pollen dispersal period, and participants with pollen allergy symptoms were not excluded during selection criteria, only those with severe allergy symptoms were excluded. Therefore, it was possible that pollen exposure influenced common cold-like symptoms observed in this study. Accordingly, a subgroup analysis excluding the 2015_Sugimura study was conducted. The analysis revealed that in the LC-Plasma group there was a tendency towards a reduction in the cumulative days of sore throat compared to the control group (MD=-0.62, 95%CI: -1.26 to 0.02, p=0.058, I^2^ = 0%; 324 participants in four studies) (**Supplementary Figure 4A**). No significant change was observed in the cumulative days of runny nose with LC-Plasma intake compared to the control group (MD=-0.67, 95%CI: -1.58 to 0.25, p=0.152, I^2^ = 0%; 206 participants in three studies) (**Supplementary Figure 4B**). LC-Plasma intake significantly reduced the cumulative days of cough per 28 days compared to the control group (MD=-0.67, 95%CI: -1.30 to -0.028, p=0.041, I^2^ = 0%; 304 participants in four studies) (**Supplementary Figure 4C**). No subgroup analysis was performed for feverishness, as it is less likely related to pollen allergy symptoms.

Common cold-like symptoms such as runny nose and sore throat are similar to symptoms of other diseases, including allergies. Thus, symptoms caused by factors other than common cold may have influenced the analyses. Although there is no standardized or universally accepted medical definition of the common cold, it is generally defined as the simultaneous occurrence of three symptoms: sore throat, runny nose or cough. Therefore, days with at least two of these three symptoms were defined as “common cold onset days” for analysis of cumulative number of days of common cold. The results showed a tendency towards a reduction in the cumulative number of cold days with LC-Plasma intake compared to the control group (MD=-0.58, 95%CI: -1.20 to 0.03, p=0.061, I^2^ = 0%; 419 participants in four studies) (**Supplementary Figure 5**).

To assess the methodological impact in the effects to common-cold like symptoms of LC-Plasma, we performed a sensitivity analysis that included all participants analysed in each study regardless of their cold status at the start of intervention. The proportion of participants with sore throat on the first day of intervention was higher in the control group in four out five studies. In the LC-Plasma group, a tendency towards a reduction in the cumulative number of days of sore throat compared to the control group (MD=-0.61, 95%CI: -1.30 to 0.07, p=0.0768, I^2^ = 0%; 556 participants in four studies)(**Supplementary Figure 6A**), while no significant change was observed in the analysis of per-protocol of MA (MD=-0.35, 95%CI: -0.90 to 0.20, p=0.209, I^2^ = 0%; 521 participants in five studies) (**Figure 3A**). For the cumulative number of days of cough, the proportion of participants with cough on the first day of intervention was higher in the LC-Plasma group in five out five studies, with a statistically significant difference observed in 2016_Fujii study. No significant change was observed in the LC-Plasma group compared to the control group (MD=-0.23, 95%CI: -1.02 to 0.55, p=0.559, I^2^ = 5%; 556 participants in five studies) (**Supplementary Figure 6C**), while LC-Plasma intake significantly reduced the cumulative number of days per 28 days with cough compared to the control group in per-protocol of MA (**Figure 3C**). No significant difference was observed with the cumulative number of days of runny nose in the result in the analysis of all participants (MD=-0.62, 95%CI: -1.57 to 0.33, p=0.199, I^2^ = 0%; 449 participants in four studies) (**Supplementary Figure 6B**), as with the result in the analysis of per-protocol of MA (MD=-0.44, 95%CI: -1.29 to 0.40, p=0.306, I^2^ = 0%; 378 participants in four studies)(**Figure 3B**). Similarly, LC-Plasma intake significantly reduced the cumulative number of days with feverishness compared to the control group in the analysis of all participants (MD=-0.28, 95%CI: -0.55 to -0.01, p=0.039, I^2^ = 0%; 461 participants in four studies)(**Supplementary Figure 6D**), which was consistent with those in per-protocol of MA (MD=-0.26, 95%CI: -0.52 to -0.0038, p=0.047, I^2^ = 0%; 456 participants in four studies) (**Figure 3D**).

#### 3.4.4 Publication and reporting bias

**Supplementary Figure 7** shows funnel plots of pDC activity and cumulative number of days of common cold symptoms. While the presence of reporting bias could not be assessed because the number of studies integrated in this analysis was limited to a maximum of six, based on the results of domain 5 in the RoB2 assessment, the risk of reporting bias was considered low (**Supplementary Figure 1**).

#### 3.4.5 Certainty of evidence

**Table 2** presents the results of the overall certainty of evidence. Since pDC activation and the cumulative number of days of common cold symptoms were continuous variables, imprecision was downgraded by one level when the sample size was less than 400. The certainty of evidence for pDC activation was rated as “moderate” based on CD86 as an indicator, and “low” based on HLA-DR as an indicator; consequently, the overall certainty of evidence for pDC activation was assessed as “moderate” (**Table 2**). For the cumulative incidence days of sore throat, cough, feverishness, and cold (defined as the occurrence of two or more symptoms among sore throat, runny nose, or cough), the certainty of evidence was rated as “low”, whereas the certainty of evidence for the cumulative incidence days of nasal discharge was rated as “very low” (**Table 2**).

**Table 2:** Certainty of evidence.

Outcome	No. of studies (no. of participants)	Risk of bias	Inconsistency	Indirectness	Imprecision	Other	Certainty (overall score)
pDC activity (Total)	**8** **(619)**	**-1**	**0**	**0**	**0**	**0**	**Moderate**
- pDC activity (CD86)	6 (511)	-1	0	0	0	0	Moderate
- pDC activity (HLA-DR)	5 (322)	-1	0	0	-1	0	Low
Cumulative number of days of common cold-like symptoms (Total)	**5** **(555)**	**-2**	**0**	**0**	**0**	**0**	**Low**
- Sore throat	5 (521)	-2	0	0	0	0	Low
- Runny nose	4 (378)	-2	0	0	-1	0	Very Low
- Cough	5 (503)	-2	0	0	0	0	Low
- Feverishness	4 (456)	-1	0	0	0	0	Low
- Common cold (≥ 2 symptoms of sore throat, runny nose and cough)	4 (419)	-2	0	0	0	0	Low

LC-Plasma, *Lactococcus lactis* strain Plasma; pDC, plasmacytoid dendritic cells; HLA-DR, Human Leukocyte Antigen - DR isotype; CD86, costimulatory molecule, Cluster of Differentiation 86 The bold values indicate the overall assessment of the primary outcome (pDC activity) and the secondary outcome (cumulative number of days with cold‑like symptoms).

## 4 Discussion

The common cold is a conventional term for a mild upper respiratory illness (5). It is reported that adults have two to five common colds each year on average and approximately 70% of individuals are reported to experience at least one episode of common cold per year (4, 5, 29, 38). Usually, the common cold has mild symptoms and resolves without treatment; however, it still causes significant economic losses due to decreased QOL, absenteeism, and reduced workforce. For example, in the United States, the common cold results in approximately 20 million days of absenteeism per year. This highlights the importance of daily preventive measures against the common cold (4, 5). Many researchers have been engaged in the research and development of food products which are effective in preventing the common cold. Nutritional components such as vitamins C and D and zinc have been reported in many studies to be effective in preventing colds and shortening their duration. Their comprehensive effects have also been examined through meta-analyses and systematic reviews (39–41). Some probiotics, including lactic acid bacteria, have also been studied for their effects on preventing viral infections such as the common cold. However, only a limited number of studies have been conducted in this area, with particularly few focusing on specific strains, meaning that comprehensive evaluations involving immune cells and colds are rare (42–46). LC-Plasma was identified as a unique strain of lactic acid bacteria that activates pDCs, and multiple studies have tested its effects on pDC activation and the prevention of cold-like symptoms (18–22, 30, 35–37). These previous studies enabled us to comprehensively verify the effects of LC-Plasma in the present study.

The meta-analysis in this study used data from 746 individuals obtained through eight RCTs. It revealed that, compared to the control group, LC-Plasma intake maintains significantly higher expression levels of the pDC activation markers CD86 and HLA-DR (CD86: SMD = 0.37, 95% CI: 0.17 to 0.57; HLA-DR: SMD = 0.63, 95% CI: 0.36 to 0.90). In addition, it identified effects on cold-like symptoms, specifically a significant reduction in the cumulative number of days with cough (MD = -0.69, 95% CI: -1.24 to -0.14) and the cumulative number of days with feverishness (MD = -0.26, 95% CI: -0.52 to -0.0038). Significant differences were not always observed within individual studies; however, the direction of the observed effects was consistent. To assess the clinical significance of the intervention effect on cold-like symptoms, we calculated weighted averages of the cumulative number of days of cough and feverishness per 28-day period using the control group data from each study (Cough: 2.02 days, Feverishness: 0.64 days). The estimated intervention effects for cumulative number of days of cough and feverishness correspond to 34.2% and 40.5%, respectively, of the weighted mean values in the control groups, suggesting that LC-Plasma intake may reduce the cumulative cough or feverishness days to approximately one-third.

Symptoms of the common cold usually begin with a sore throat, followed by a runny nose and cough. While sore throat resolves quickly, the cough is often persistent, and secondary infection with a virus or other agents may cause the cough to last for three weeks or longer (2, 6, 28, 47). Fever is considered a more severe symptom and is regarded as an influenza-like symptom. Thus, fever is considered one of the indicators reflecting the severity and seriousness of common cold cases (2). This study found that LC-Plasma intake significantly reduced the cumulative number of days with cough and feverishness. Thus, it suggests the possibility that LC-Plasma intake suppresses the progression of post-infection cold-like symptoms by inhibiting the proliferation of viruses and other agents after infection.

By conducting an IPD meta-analysis, the present study was able to comprehensively verify the effect of LC-Plasma intervention on pDC activation, using the change observed before and after the intervention as an indicator. Common cold-like symptoms were evaluated differently among previous studies; the present IPD meta-analysis applied a uniform standard and successfully obtained integrated results with low heterogeneity. Previous studies assessed cold-like symptoms by recording the occurrence of individual symptoms each day. In contrast, the present study defined a symptom as expressed when it appeared for three consecutive days. This approach allowed us to perform an analysis that better reflects the characteristics of the common cold, which is a strength of the present study. Furthermore, previous studies calculated the total cumulative number of days with symptoms for each group and then compared the groups. In contrast, the present study used IPD to calculate the cumulative number of days with cold-like symptoms and the average scores for individual participants. It then obtained the mean values and SD for each group, followed by group comparisons. This method allowed us to perform an analysis that takes individual variations into account.

Meanwhile, the present study has several limitations. The first limitation concerns the evidence base. Of the eight previous studies selected for this study, seven were conducted in Japan and one in Malaysia. In addition, all included studies enrolled healthy adults only. Therefore, this study does not include verification of the effects of LC-Plasma in non-Asian individuals, elderly adults, children, or immunocompromised individuals. The second limitation is the inability to quantitatively and objectively assess actual fever. Although this study demonstrated that LC-Plasma intake significantly reduced the cumulative number of days with “feverishness”, this is a subjectively evaluated indicator. Tests that use body temperature as an evaluation index will be necessary to verify the effects of LC-Plasma intake on fever in more detail.

Furthermore, for the analysis of cold-like symptoms, the present study may have adopted papers with a high risk of bias, resulting in the overall certainty of evidence for cold-like symptoms being rated as “low”. The high risk of bias was due to the papers not providing detailed information on test conditions such as randomization, blinding, and allocation concealment. Also, in order to verify the effects of LC-Plasma on preventing the common cold, the meta-analysis of the present study excluded participants who exhibited cold-like symptoms on the intervention start date, which resulted in a high-risk rating in domain 2. In a sensitivity analysis that included all participants analysed in each study, the cumulative number of days of cough, which had shown a significant difference in the per-protocol of MA, no longer differed significantly between the two groups, whereas the cumulative number of days of sore throat, which was not significant in the per-protocol of MA, showed a trend toward reduction in the LC-Plasma group. These discrepancies across analytical conditions may reflect an influence of between-group imbalances in the number of participants with cold-like symptoms at start of intervention on the effect estimates. Future studies with low risk of bias that enroll only participants without common cold-like symptoms at baseline, and that are appropriately designed, implemented, and reported are expected to provide stronger evidence supporting the effect of LC-Plasma. Among the common cold-like symptoms, the certainty of evidence for runny nose was rated as “very low”. The likely cause of this was the small sample size, due to the scarce availability of previous studies reporting runny nose. Thus, we believe it is necessary to verify this through additional RCTs. Furthermore, among the selected studies, three conducted interventions during the cedar pollen dispersal season. Therefore, the pDC activation levels and cold-like symptoms, which were the evaluation indices in these studies, may have been affected by allergic symptoms. In fact, a study (2015_Sugimura) that conducted the intervention during the pollen dispersal season without excluding all individuals with allergic symptoms to pollen reported a higher cumulative number of days with runny nose compared to other studies. In the subgroup analysis conducted without the 2015_Sugimura study, LC-Plasma tended to reduce the cumulative number of days with sore throat and runny nose compared to the overall analysis. In addition, since all the papers used for the present study included authors who were employees of Kirin Company, Limited (the former name of Kirin Holdings Company, Limited) or Kirin Holdings Company, Limited, the possibility of a conflict of interest cannot be ruled out.

A further limitation in the study process is that we were unable to obtain IPD from the study with the largest sample size (2016_Shibata) among the selected studies. As a result, the analysis of pDC activation in this specific study had to be performed using estimated values, and we were unable to integrate data for cold-like symptoms. If the data from the 2016_Shibata study were integrated with IPD, the overall results could be significantly affected. In fact, in the analysis of pDC activation (HLA-DR), the subgroup analysis that excluded data from the 2016_Shibata study showed a substantial decrease in heterogeneity. This suggests that the 2016_Shibata study was the source of heterogeneity. Moreover, since the common cold does not have a clear medical definition, the present study defined the onset of cold-like symptoms as the presence of characteristic symptoms of the common cold for three consecutive days. The appropriateness of this definition also requires further validation. Since the present study does not take into account symptoms other than sore throat, runny nose, or feverishness, analyses based on other subjective symptoms should also be considered.

## 5 Conclusion

This IPD meta-analysis is the first integrated analysis demonstrating that daily intake of LC-Plasma maintains high pDC activity and reduces the cumulative duration of cough and feverishness using identical analytical conditions for common cold-like symptoms, suggesting its potential benefit in the reducing cold-like symptoms. However, because statistically significant effects were observed only for cough and feverishness, the overall effect size for “total common cold-cold like symptoms” should be interpreted cautiously due to the high risk of bias. To further validate the effects of LC-Plasma on preventing common cold-like symptoms, future intervention trials with appropriately calculated sample sizes, optimal timing of long-term intervention, and carefully defined participant without cold-like symptoms at baseline and analysis populations based on these findings are warranted. Additionally, new intervention studies evaluating the incidence of colds through physician diagnosis rather than subjective assessment are expected.

## Data Availability

The raw data supporting the conclusions of this article will be made available by the authors, without undue reservation.

## References

[1] Hemilä H . Vitamin C and infections. Nutrients. (2017) 9:339. doi: 10.3390/nu9040339, PMID: 28353648 PMC5409678

[2] Eccles R . Common cold. Front Allergy. (2023) 4:1224988. doi: 10.3389/falgy.2023.1224988, PMID: 37426629 PMC10324571

[3] Tobin EH Thomas M Bomar PA . Upper respiratory tract infections with focus on the common cold. In: StatPearls. StatPearls Publishing, Treasure Island (FL (2025). Available online at: http://www.ncbi.nlm.nih.gov/books/NBK532961., PMID: 30422556

[4] Fendrick AM Monto AS Nightengale B Sarnes M . The economic burden of non-influenza-related viral respiratory tract infection in the United States. Arch Intern Med. (2003) 163:487–94. doi: 10.1001/archinte.163.4.487, PMID: 12588210

[5] Heikkinen T Järvinen A . The common cold. Lancet. (2003) 361:51–9. doi: 10.1016/S0140-6736(03)12162-9, PMID: 12517470 PMC7112468

[6] Eccles R . Understanding the symptoms of the common cold and influenza. Lancet Infect Dis. (2005) 5:718–25. doi: 10.1016/S1473-3099(05)70270-X, PMID: 16253889 PMC7185637

[7] Reizis B . Plasmacytoid dendritic cells: development, regulation, and function. Immunity. (2019) 50:37–50. doi: 10.1016/j.immuni.2018.12.027, PMID: 30650380 PMC6342491

[8] Swiecki M Colonna M . The multifaceted biology of plasmacytoid dendritic cells. Nat Rev Immunol. (2015) 15:471–85. doi: 10.1038/nri3865, PMID: 26160613 PMC4808588

[9] Colonna M Trinchieri G Liu Y-J . Plasmacytoid dendritic cells in immunity. Nat Immunol. (2004) 5:1219–26. doi: 10.1038/ni1141, PMID: 15549123

[10] Ito T Amakawa R Inaba M Ikehara S Inaba K Fukuhara S . Differential regulation of human blood dendritic cell subsets by IFNs. J Immunol. (2001) 166:2961–9. doi: 10.4049/jimmunol.166.5.2961, PMID: 11207245

[11] Kadowaki N Ho S Antonenko S Malefyt RW Kastelein RA Bazan F . Subsets of human dendritic cell precursors express different toll-like receptors and respond to different microbial antigens. J Exp Med. (2001) 194:863–9. doi: 10.1084/jem.194.6.863, PMID: 11561001 PMC2195968

[12] Siegal FP Kadowaki N Shodell M Fitzgerald-Bocarsly PA Shah K Ho S . The nature of the principal type 1 interferon-producing cells in human blood. Science. (1999) 284:1835–7. doi: 10.1126/science.284.5421.1835, PMID: 10364556

[13] Gilliet M Cao W Liu Y-J . Plasmacytoid dendritic cells: sensing nucleic acids in viral infection and autoimmune diseases. Nat Rev Immunol. (2008) 8:594–606. doi: 10.1038/nri2358, PMID: 18641647

[14] Sadler AJ Williams BRG . Interferon-inducible antiviral effectors. Nat Rev Immunol. (2008) 8:559–68. doi: 10.1038/nri2314, PMID: 18575461 PMC2522268

[15] Kanauchi O Low ZX Jounai K Tsuji R AbuBakar S . Overview of anti-viral effects of probiotics via immune cells in pre-, mid- and post-SARS-CoV2 era. Front Immunol. (2023) 14:1280680. doi: 10.3389/fimmu.2023.1280680, PMID: 38116008 PMC10728489

[16] Banchereau J Steinman RM . Dendritic cells and the control of immunity. Nature. (1998) 392:245–52. doi: 10.1038/32588, PMID: 9521319

[17] Tavano B Boasso A . Effect of immunoglobin-like transcript 7 cross-linking on plasmacytoid dendritic cells differentiation into antigen-presenting cells. PloS One. (2014) 9:e89414. doi: 10.1371/journal.pone.0089414, PMID: 24586760 PMC3929723

[18] Jounai K Ikado K Sugimura T Ano Y Braun J Fujiwara D . Spherical lactic acid bacteria activate plasmacytoid dendritic cells immunomodulatory function via TLR9-dependent crosstalk with myeloid dendritic cells. PloS One. (2012) 7:e32588. doi: 10.1371/journal.pone.0032588, PMID: 22505996 PMC3323594

[19] Sugimura T Jounai K Ohshio K Tanaka T Suwa M Fujiwara D . Immunomodulatory effect of Lactococcus lactis JCM5805 on human plasmacytoid dendritic cells. Clin Immunol. (2013) 149:509–18. doi: 10.1016/j.clim.2013.10.007, PMID: 24239838

[20] Sugimura T Takahashi H Jounai K Ohshio K Kanayama M Tazumi K . Effects of oral intake of plasmacytoid dendritic cells-stimulative lactic acid bacterial strain on pathogenesis of influenza-like illness and immunological response to influenza virus. Br J Nutr. (2015) 114:727–33. doi: 10.1017/S0007114515002408, PMID: 26234407

[21] Suzuki H Kanayama M Fujii T Fujiwara D Sugimura H . Effects of the Beverage Containing Lactococcus lactis subsp. lactis JCM5805 on Antiviral Immune Responses and Maintenance of Physical Conditions—A Randomized, Double-Blind, Placebo-Controlled, Parallel-Group Trial-. Jpn Pharmacol Ther. (2015) 43:1465–72. Available online at: https://www.pieronline.jp/content/article/0386-3603/43100/1465.

[22] Shibata T Kanayama M Haida M Fujimoto S Oroguchi T Sata K . Lactococcus lactis JCM5805 activates anti-viral immunity and reduces symptoms of common cold and influenza in healthy adults in a randomized controlled trial. J Funct Foods. (2016) 24:492–500. doi: 10.1016/j.jff.2016.03.035

[23] Stewart LA Clarke M Rovers M Riley RD Simmonds M Stewart G . Preferred reporting items for a systematic review and meta-analysis of individual participant data: the PRISMA-IPD statement. JAMA. (2015) 313:1657–65. doi: 10.1001/jama.2015.3656, PMID: 25919529

[24] Page MJ McKenzie JE Bossuyt PM Boutron I Hoffmann TC Mulrow CD . The PRISMA 2020 statement: an updated guideline for reporting systematic reviews. BMJ. (2021) 372:n71. doi: 10.1136/bmj.n71, PMID: 33782057 PMC8005924

[25] Sterne JAC Savović J Page MJ Elbers RG Blencowe NS Boutron I . RoB 2: a revised tool for assessing risk of bias in randomised trials. BMJ. (2019) 366:l4898. doi: 10.1136/bmj.l4898, PMID: 31462531

[26] Shimada M . Differential diagnosis of common cold. J Japan Soc Infection Aerosol Otorhinolaryngology. (2020) 8:172–75. doi: 10.24805/jjsiao.8.3_172

[27] Shieh W-J . Human adenovirus infections in pediatric population - An update on clinico–pathologic correlation. Biomed J. (2022) 45:38–49. doi: 10.1016/j.bj.2021.08.009, PMID: 34506970 PMC9133246

[28] Tokuda H . How to treat Acute Respiratory Infection in the elderly and those with chronic lung comorbidity. Jpn Open J Respir Med. (2018) 2:e00065. doi: 10.24557/kokyurinsho.2.e00065

[29] Turner RB . Epidemiology, pathogenesis, and treatment of the common cold. Ann Allergy Asthma Immunol. (1997) 78:531–40. doi: 10.1016/S1081-1206(10)63213-9, PMID: 9207716 PMC7128171

[30] Fujii T Jounai K Horie A Takahashi H Suzuki H Ohshio K . Effects of heat-killed *Lactococcus lactis* subsp. *lactis* JCM 5805 on mucosal and systemic immune parameters, and antiviral reactions to influenza virus in healthy adults; a randomized controlled double-blind study. J Funct Foods. (2017) 35:513–21. doi: 10.1016/j.jff.2017.06.011

[31] Satomura K Kitamura T Kawamura T Shimbo T Watanabe M Kamei M . Prevention of upper respiratory tract infections by garglingA randomized trial. Am J Prev Med. (2005) 29:302–7. doi: 10.1016/j.amepre.2005.06.013, PMID: 16242593

[32] Kanda Y . Investigation of the freely available easy-to-use software ‘EZR’ for medical statistics. Bone Marrow Transplant. (2013) 48:452–8. doi: 10.1038/bmt.2012.244, PMID: 23208313 PMC3590441

[33] Cochrane Handbook for Systematic Reviews of Interventions (current version) Version 6.5 (2024). Available online at: https://www.cochrane.org/authors/handbooks-and-manuals/handbook/current (Accessed August 26, 2025).

[34] Guyatt GH Oxman AD Vist GE Kunz R Falck-Ytter Y Alonso-Coello P . GRADE: an emerging consensus on rating quality of evidence and strength of recommendations. BMJ. (2008) 336:924–6. doi: 10.1136/bmj.39489.470347.AD, PMID: 18436948 PMC2335261

[35] Komano Y Shimada K Naito H Fukao K Ishihara Y Fujii T . Efficacy of heat-killed Lactococcus lactis JCM 5805 on immunity and fatigue during consecutive high intensity exercise in male athletes: a randomized, placebo-controlled, double-blinded trial. J Int Soc Sports Nutr. (2018) 15:39. doi: 10.1186/s12970-018-0244-9, PMID: 30071871 PMC6090876

[36] Khor C-S Tsuji R Lee H-Y Nor’e S-S Sahimin N Azman A-S . Lactococcus lactis strain plasma intake suppresses the incidence of dengue fever-like symptoms in healthy Malaysians: A randomized, double-blind, placebo-controlled trial. Nutrients. (2021) 13:4507. doi: 10.3390/nu13124507, PMID: 34960061 PMC8707015

[37] Komano Y Fukao K Shimada K Naito H Ishihara Y Fujii T . Effects of Ingesting Food Containing Heat-Killed Lactococcus lactis Strain Plasma on Fatigue and Immune-Related Indices after High Training Load: A Randomized, Double-Blind, Placebo-Controlled, and Parallel-Group Study. Nutrients. (2023) 15:1754. doi: 10.3390/nu15071754, PMID: 37049594 PMC10096552

[38] MontoAS . Epidemiology of viral respiratory infections. Am J Med. (2002) 112 Suppl 6A:4S–12S. doi: 10.1016/s0002-9343(01)01058-0, PMID: 11955454

[39] Science M Johnstone J Roth DE Guyatt G Loeb M . Zinc for the treatment of the common cold: a systematic review and meta-analysis of randomized controlled trials. CMAJ. (2012) 184:E551–561. doi: 10.1503/cmaj.111990, PMID: 22566526 PMC3394849

[40] Martineau AR Jolliffe DA Hooper RL Greenberg L Aloia JF Bergman P . Vitamin D supplementation to prevent acute respiratory tract infections: systematic review and meta-analysis of individual participant data. BMJ. (2017) 356:i6583. doi: 10.1136/bmj.i6583, PMID: 28202713 PMC5310969

[41] Ran L Zhao W Wang H Zhao Y Bu H . Vitamin C as a supplementary therapy in relieving symptoms of the common cold: A meta-analysis of 10 randomized controlled trials. BioMed Res Int. (2020) 2020:8573742. doi: 10.1155/2020/8573742, PMID: 33102597 PMC7569434

[42] de Vrese M Winkler P Rautenberg P Harder T Noah C Laue C . Probiotic bacteria reduced duration and severity but not the incidence of common cold episodes in a double blind, randomized, controlled trial. Vaccine. (2006) 24:6670–4. doi: 10.1016/j.vaccine.2006.05.048, PMID: 16844267

[43] Liu S Hu P Du X Zhou T Pei X . Lactobacillus rhamnosus GG supplementation for preventing respiratory infections in children: a meta-analysis of randomized, placebo-controlled trials. Indian Pediatr. (2013) 50:377–81. doi: 10.1007/s13312-013-0123-z, PMID: 23665598

[44] Shida K Sato T Iizuka R Hoshi R Watanabe O Igarashi T . Daily intake of fermented milk with Lactobacillus casei strain Shirota reduces the incidence and duration of upper respiratory tract infections in healthy middle-aged office workers. Eur J Nutr. (2017) 56:45–53. doi: 10.1007/s00394-015-1056-1, PMID: 26419583 PMC5290054

[45] Amaral MA Guedes GHBF Epifanio M Wagner MB Jones MH Mattiello R . Network meta-analysis of probiotics to prevent respiratory infections in children and adolescents. Pediatr Pulmonol. (2017) 52:833–43. doi: 10.1002/ppul.23643, PMID: 28052594

[46] Zhao Y Dong BR Hao Q . Probiotics for preventing acute upper respiratory tract infections. Cochrane Database Syst Rev. (2022) 8:CD006895. doi: 10.1002/14651858.CD006895.pub4. Available online at: https://www.racgp.org.au/getattachment/f8f8cf6b-f7c0-47a0-8f5c-29b3a16bd0a9/attachment.aspx., PMID: 36001877 PMC9400717

[47] Jones BF Stewart MA . Duration of cough in acute upper respiratory tract infections. Aust Fam Physician. (2002) 31:971–3. 12404840

