# Supplementary material for: Lactococcus lactis strain Plasma activates plasmacytoid dendritic cells and mitigates common cold-like symptoms in healthy adults: a meta-analysis of individual participant data
Source: Front Immunol. 2025 Nov 6;16:1696989. doi: 10.3389/fimmu.2025.1696989 (PMC12631266; doi:10.3389/fimmu.2025.1696989)
Supplement: Supplementary file 1 [file DataSheet1.docx]

**Supplementary Table 1. Search strategy and search terms**

PubMed

| # | Search strategy | Number of Articles |
| --- | --- | --- |
| 1 | "JCM 5805" OR "JCM5805" OR "strain Plasma" OR "strain plasma" OR "ATCC 19435" OR "ATCC 9936" OR "BCC 49730" OR "BCRC 12312" OR "CCM 1877" OR "CCUG 32211" OR "CCUG 7980" OR "CECT 185" OR "CIP 70.56" OR "DSM 20481" OR "HAMBI 1591" OR "KCTC 3769" OR "LMG 6890" OR "NBIMCC 4000" OR "NBRC 100933" OR "NCAIM B.02070" OR "NCDO 604" OR "NCIMB 6681" OR "NCTC 6681" OR "NRIC 1149" OR "VKM B-1662" OR "VTT E-90395"  *Search field was set to [Title/Abstract]. | 86 |

Cochran Library

| # | Search strategy | Number of Articles |
| --- | --- | --- |
| 1 | "JCM 5805" OR "JCM5805" OR "strain Plasma" OR "strain plasma" OR "ATCC 19435" OR "ATCC 9936" OR "BCC 49730" OR "BCRC 12312" OR "CCM 1877" OR "CCUG 32211" OR "CCUG 7980" OR "CECT 185" OR "CIP 70.56" OR "DSM 20481" OR "HAMBI 1591" OR "KCTC 3769" OR "LMG 6890" OR "NBIMCC 4000" OR "NBRC 100933" OR "NCAIM B.02070" OR "NCDO 604" OR "NCIMB 6681" OR "NCTC 6681" OR "NRIC 1149" OR "VKM B-1662" OR "VTT E-90395"  * Search field was set to [ti,ab,kw]. | 17 |

J-DreamIII

| # | Search strategy | Number of Articles |
| --- | --- | --- |
| 1 | "JCM 5805" OR "JCM5805" OR "LC-Plasma (in Japanese)" OR "strain Plasma" OR "strain plasma" OR "ATCC 19435" OR "ATCC 9936" OR "BCC 49730" OR "BCRC 12312" OR "CCM 1877" OR "CCUG 32211" OR "CCUG 7980" OR "CECT 185" OR "CIP 70.56" OR "DSM 20481" OR "HAMBI 1591" OR "KCTC 3769" OR "LMG 6890" OR "NBIMCC 4000" OR "NBRC 100933" OR "NCAIM B.02070" OR "NCDO 604" OR "NCIMB 6681" OR "NCTC 6681" OR "NRIC 1149" OR "VKM B-1662" OR "VTT E-90395"  *Searched by entering the above search terms in a free word search. | 123 |

UMIN-CTR

| # | Search strategy | Number of Articles |
| --- | --- | --- |
| 1 | "JCM 5805" OR "JCM5805" OR "LC-Plasma(*in Japanese*)" OR "strain Plasma" OR "strain plasma" OR "ATCC 19435" OR "ATCC 9936" OR "BCC 49730" OR "BCRC 12312" OR "CCM 1877" OR "CCUG 32211" OR "CCUG 7980" OR "CECT 185" OR "CIP 70.56" OR "DSM 20481" OR "HAMBI 1591" OR "KCTC 3769" OR "LMG 6890" OR "NBIMCC 4000" OR "NBRC 100933" OR "NCAIM B.02070" OR "NCDO 604" OR "NCIMB 6681" OR "NCTC 6681" OR "NRIC 1149" OR "VKM B-1662" OR "VTT E-90395"  *Searched by entering the above search terms in key words search. | 0 |

ICTRP

| # | Search strategy | Number of Articles |
| --- | --- | --- |
| 1 | "JCM 5805" OR "JCM5805" OR "strain Plasma" OR "strain plasma" OR "ATCC 19435" OR "ATCC 9936" OR "BCC 49730" OR "BCRC 12312" OR "CCM 1877" OR "CCUG 32211" OR "CCUG 7980" OR "CECT 185" OR "CIP 70.56" OR "DSM 20481" OR "HAMBI 1591" OR "KCTC 3769" OR "LMG 6890" OR "NBIMCC 4000" OR "NBRC 100933" OR "NCAIM B.02070" OR "NCDO 604" OR "NCIMB 6681" OR "NCTC 6681" OR "NRIC 1149" OR "VKM B-1662" OR "VTT E-90395"  *Searched by entering the above search terms in a free word search. | 2 |

**Supplementary Figure 1. Risk of bias assessment**

**(A) CD86 expression on pDC**

**
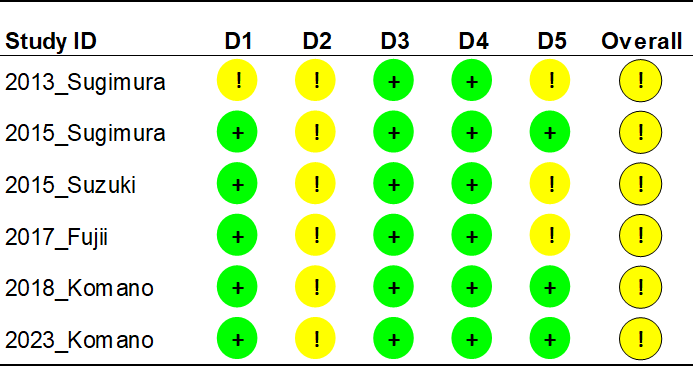
**
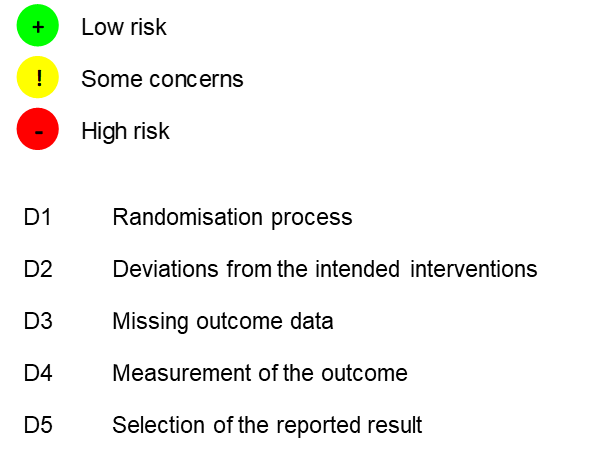


**(B) HLA-DR expression on pDC**

**(C) Sore throat**

**(D) Runny nose**

**(E) Cough**

**(F) Feverishness**

**(G) Common cold (≥ 2 symptoms of sore throat, runny nose or cough)**

**Supplementary Figure 2. IPD meta-analysis of pDC activity (HLA-DR) without 2016_Shibata study**

**
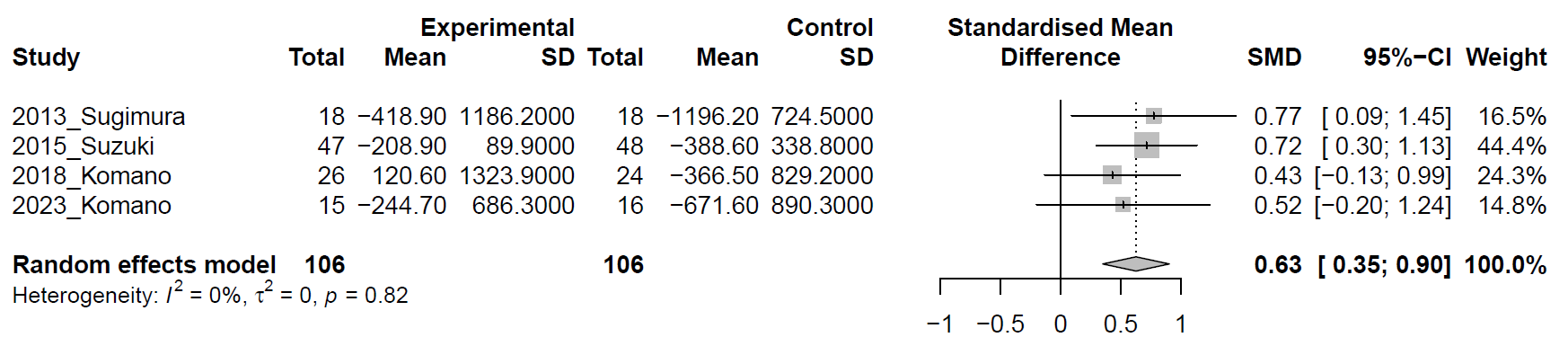
**

**Supplementary Figure 3. Forest plots of meta-analysis of severity of common cold-like symptoms**

1. **Sore throat**


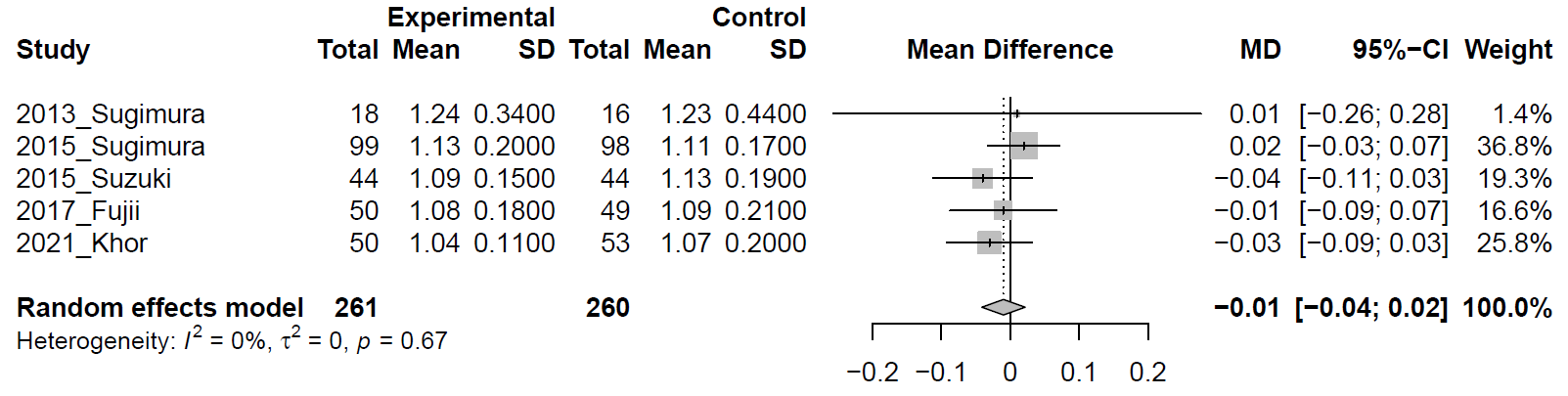


1. **Runny nose**

**
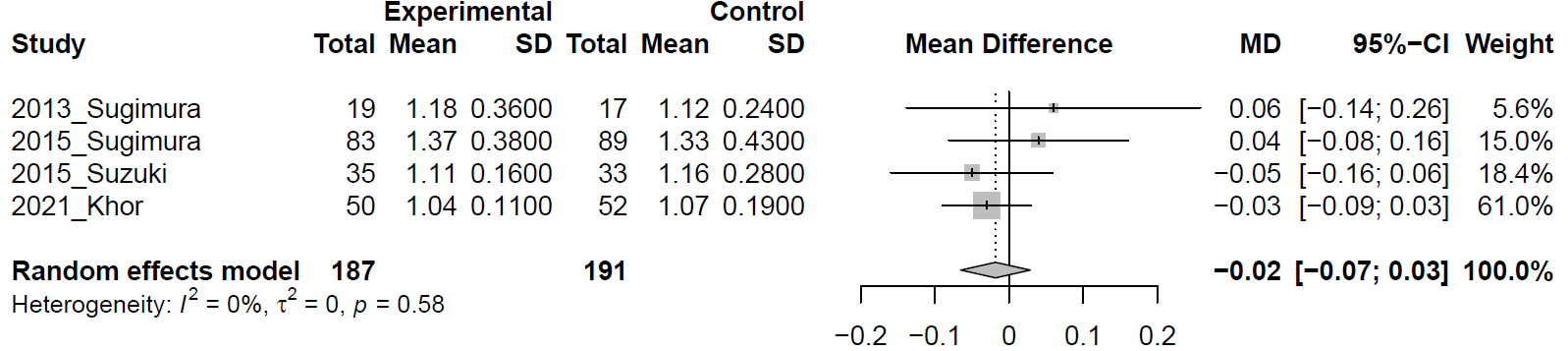
**

1. **Cough**

**
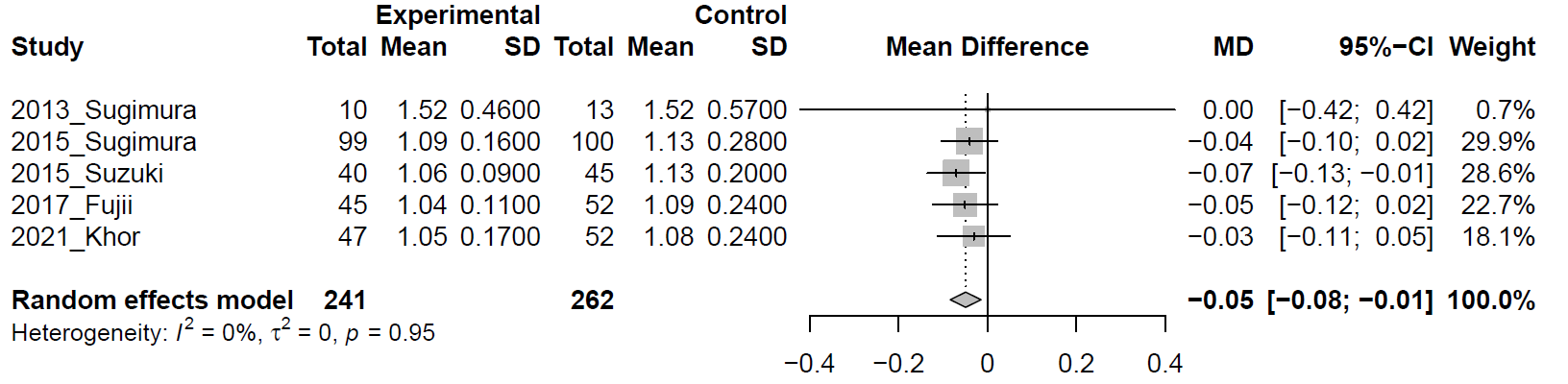
**

1. **Feverishness**

**
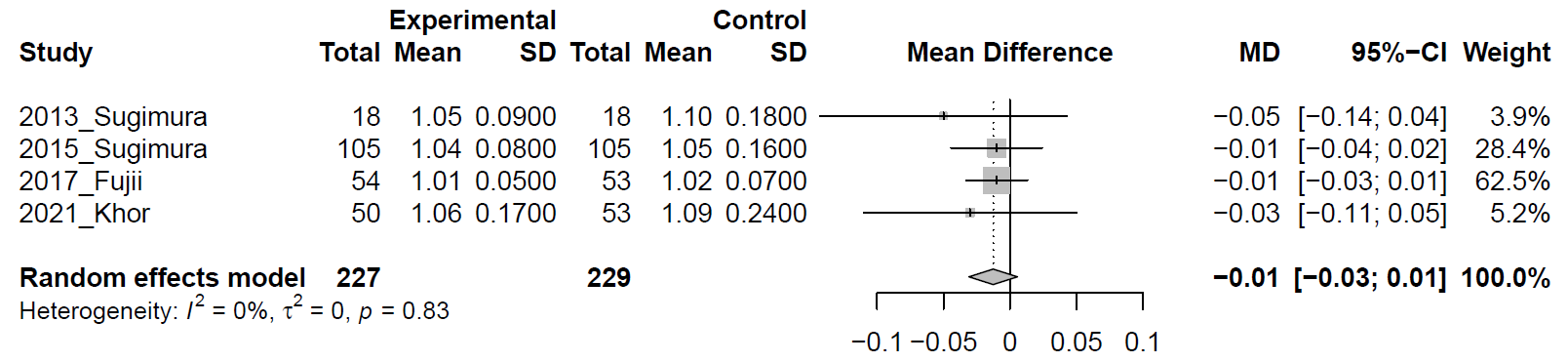
**

**Supplementary Figure 4. Forest plots of meta-analysis of cumulative number of days of common cold symptoms without 2015_Sugimura study**

1. **Sore throat**

**
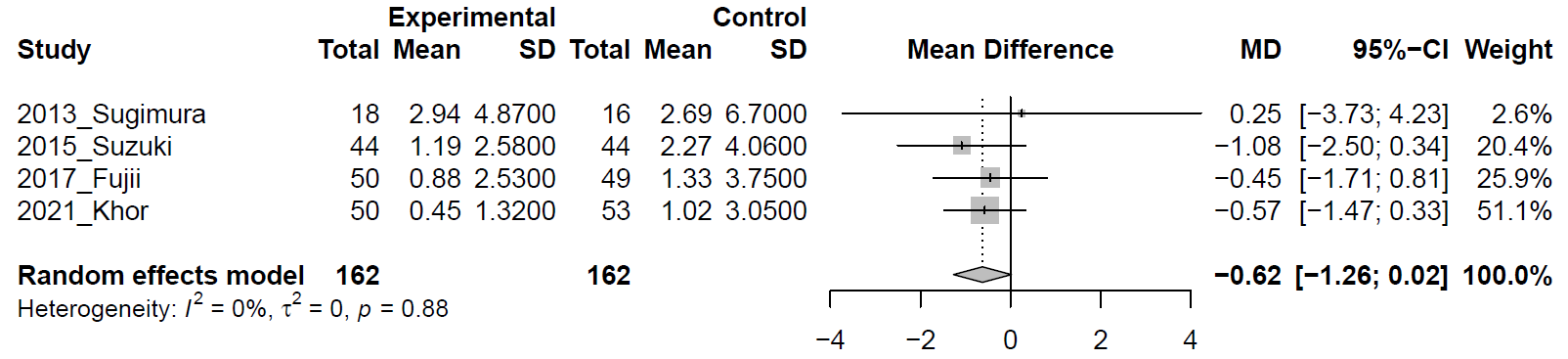
**

1. **Runny nose**

**
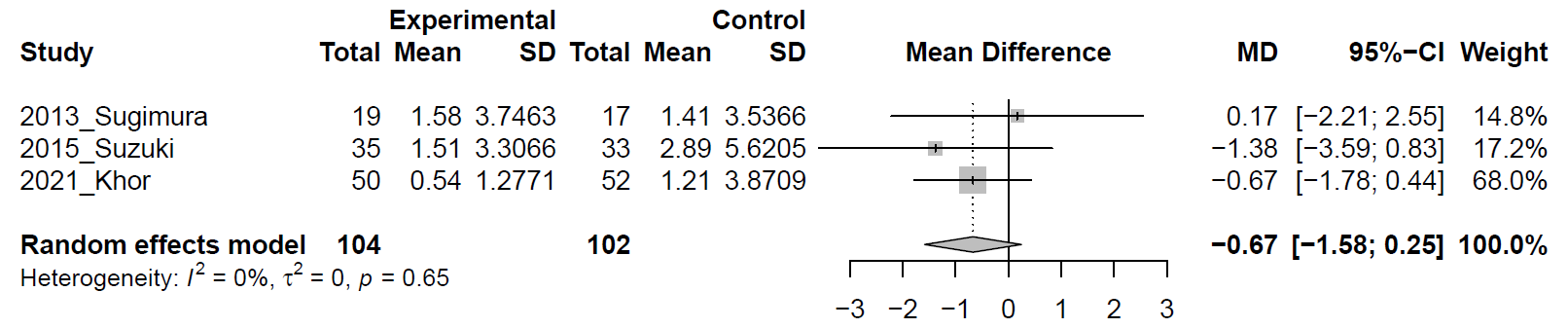
**

1. **Cough**

**
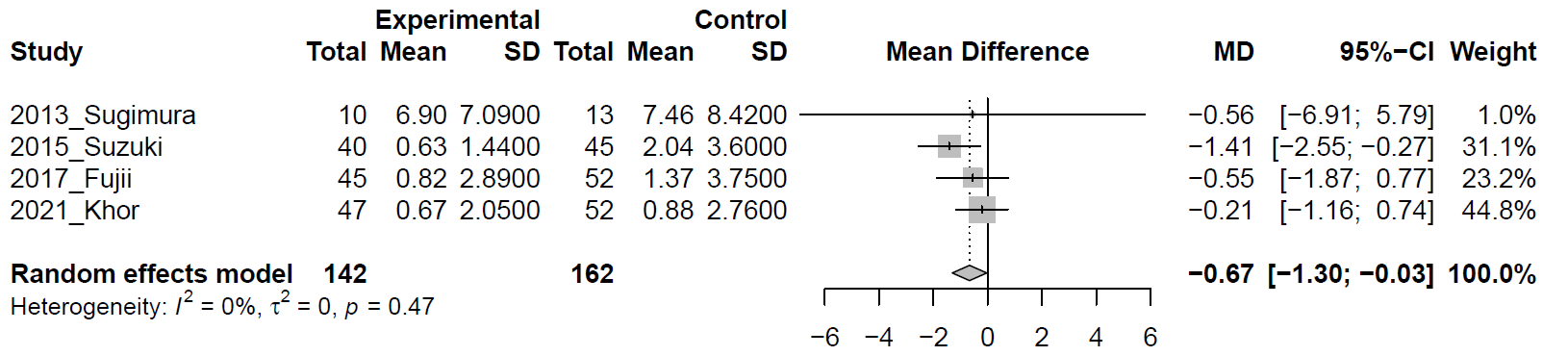
**

**Supplementary Figure 5. Forest plots of meta-analysis of cumulative number of days of common cold (≥ 2 symptoms of sore throat, runny nose or cough)**

**
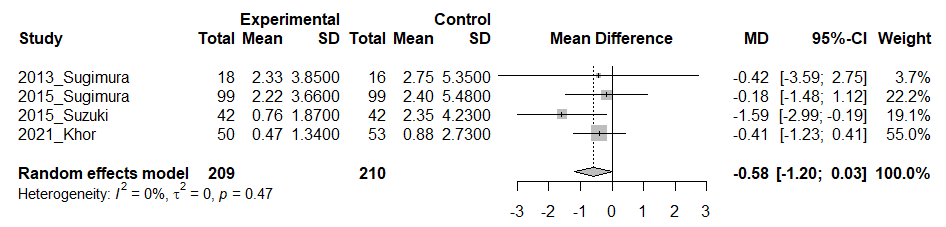
**

**Supplementary Figure 6. Forest plots of meta-analysis including participants with cold-like symptoms at intervention onset: cumulative number of days of common cold-like symptoms**

1. **Sore throat**

**
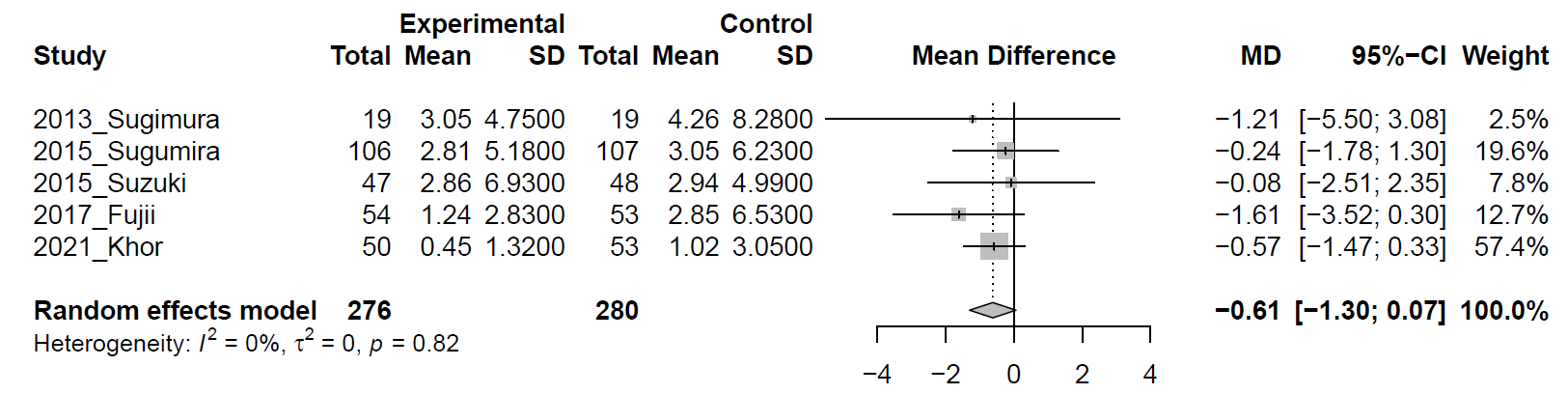
**

1. **Runny nose**

**
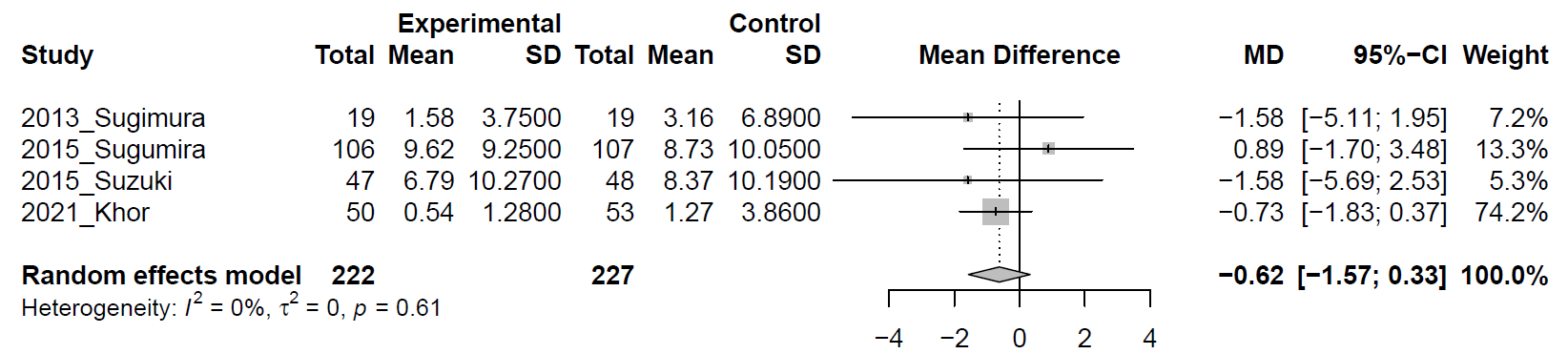
**

1. **Cough**

**
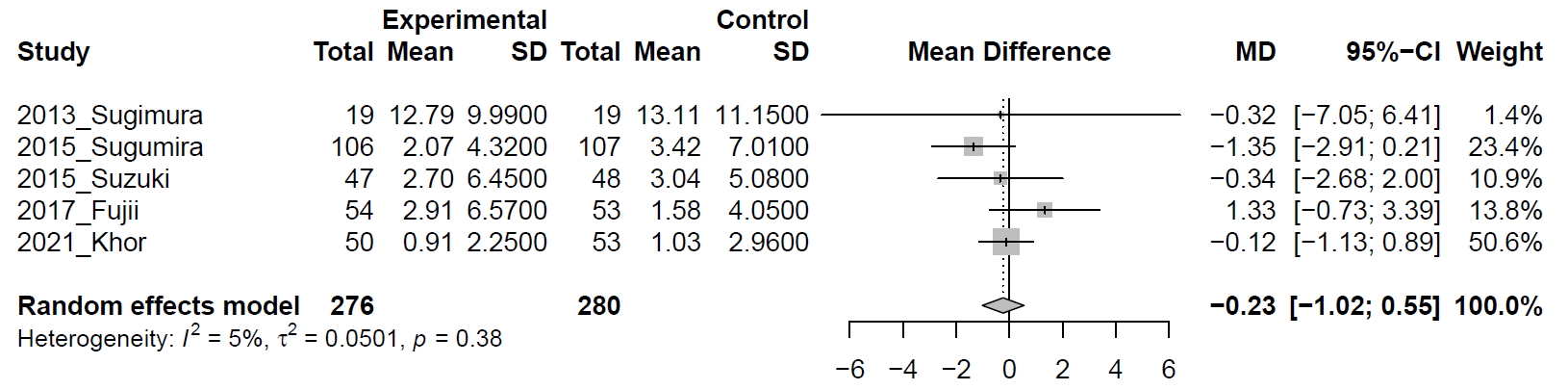
**

1. **Feverishness**

**
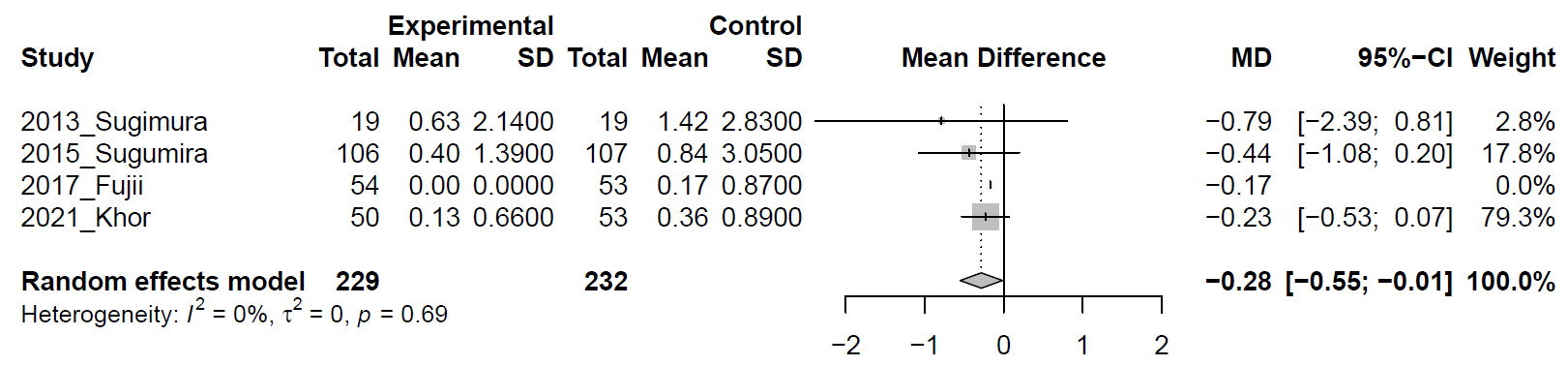
**

1. **Common cold (≥ 2 symptoms of sore throat, runny nose or cough)**

**
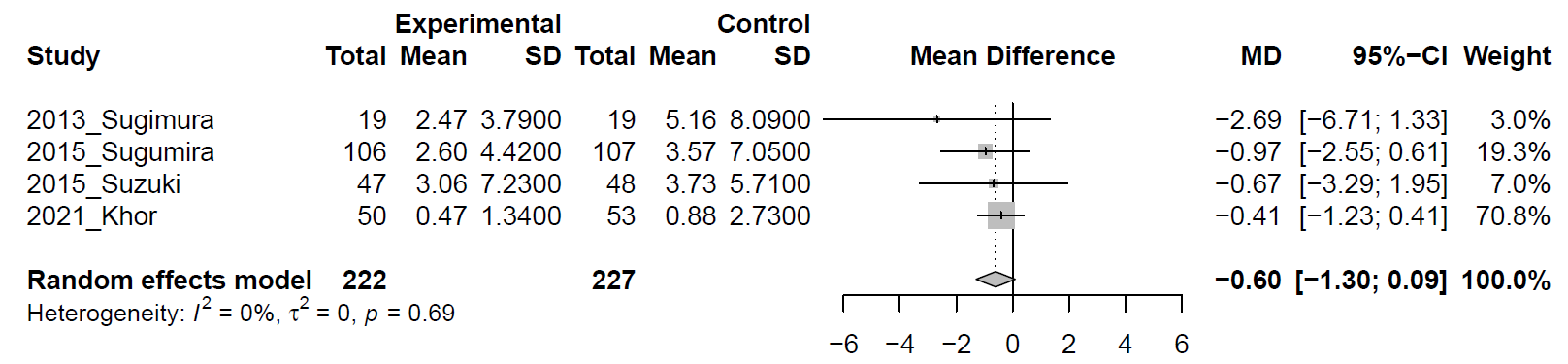
**

**Supplementary Figure 7. Funnel plots**

1. **CD86 expression on pDC　　　　　　　(B) HLA-DR expression on pDC**

**(C) Sore throat (D) Runny nose**


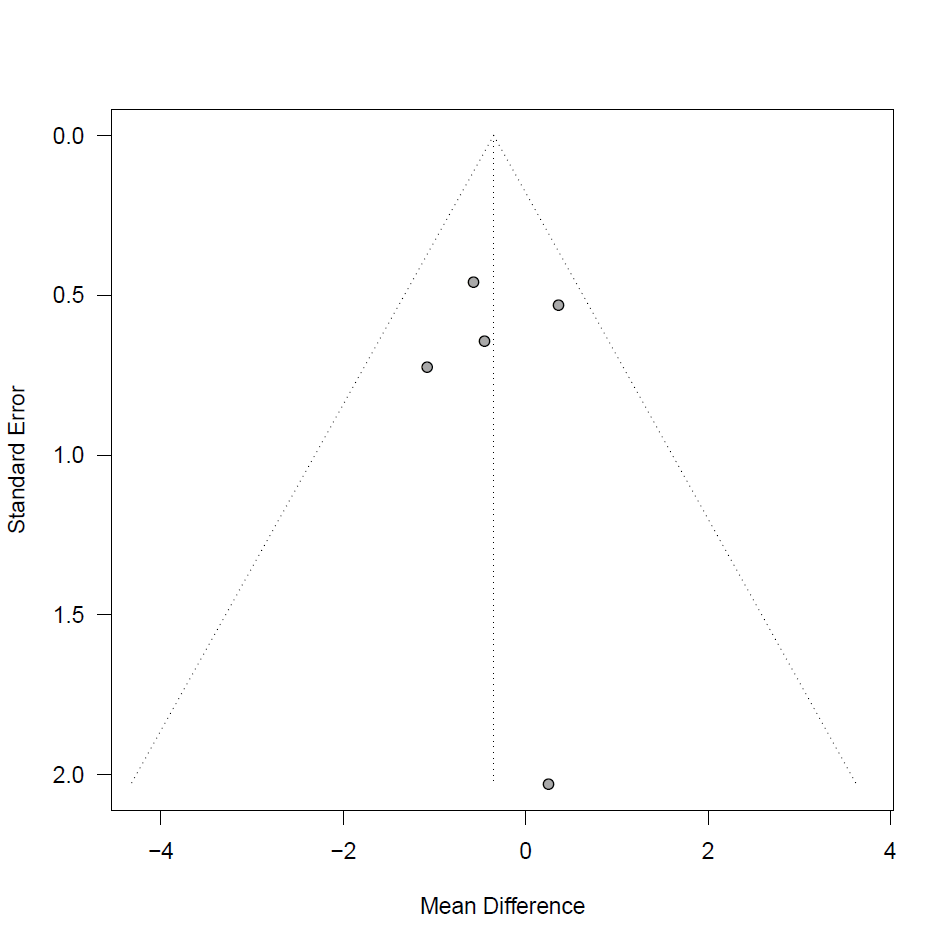
 ****

1. **Cough (F) Feverishness**


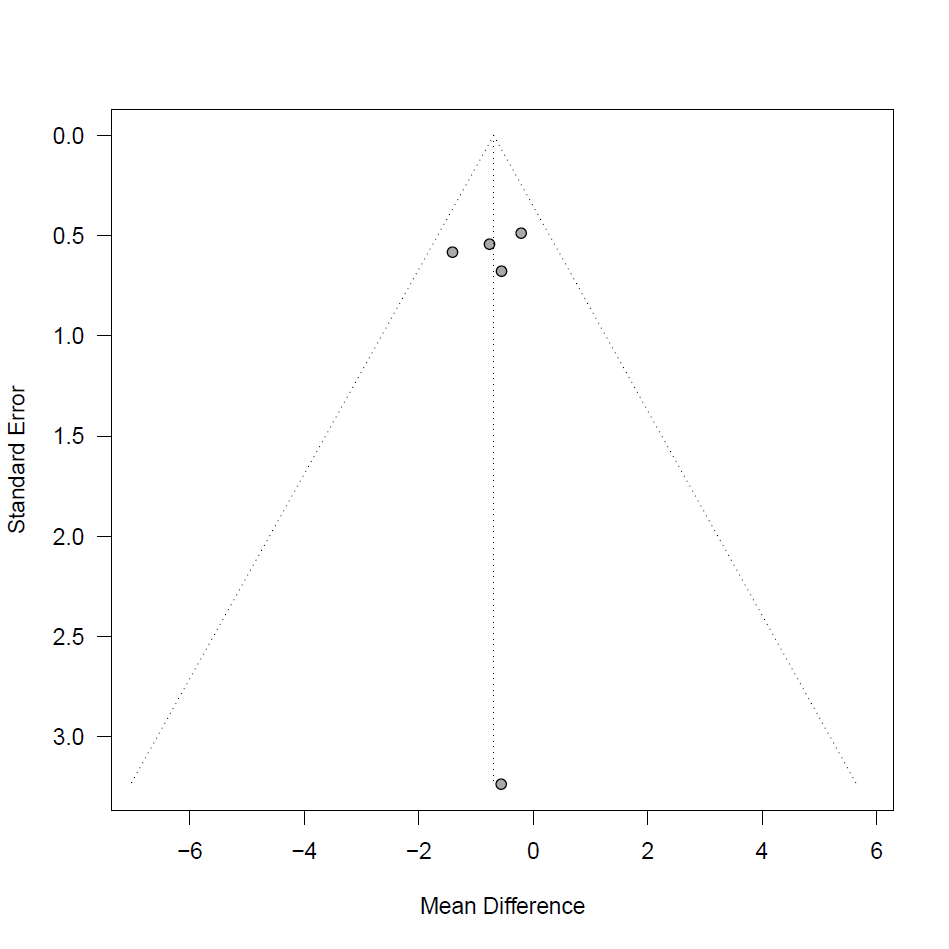
 ****

LC-Plasma: *Lactococcus lactis* strain Plasma; pDC: plasmacytoid dendritic cells; HLA-DR: Human Leukocyte Antigen - DR isotype; CD86: costimulatory molecule, Cluster of Differentiation 86
